# Supplementary material for: Medical education and distrust modulate the response of insular-cingulate network and ventral striatum in pain diagnosis
Source: eLife. 2021 Apr 27;10:e63272. doi: 10.7554/eLife.63272 (PMC8104963; doi:10.7554/eLife.63272)
Supplement: Transparent reporting form [file elife-63272-transrepform.pdf]

Westbrook Centre, Milton Road
Cambridge CB4 1YG
UK
P 01223 855340
W elifesciences.org
T @elife

eLife's transparent reporting form

We encourage authors to provide detailed information *within their submission* to facilitate the interpretation and replication of experiments. Authors can upload supporting documentation to indicate the use of appropriate reporting guidelines for health-related research (see EQUATOR Network), life science research (see the BioSharing Information Resource), or the ARRIVE guidelines for reporting work involving animal research. Where applicable, authors should refer to any relevant reporting standards documents in this form.

If you have any questions, please consult our Journal Policies and/or contact us: editorial@elifesciences.org.

Sample-size estimation

- You should state whether an appropriate sample size was computed when the study was being designed
- You should state the statistical method of sample size computation and any required assumptions
- If no explicit power analysis was used, you should describe how you decided what sample (replicate) size (number) to use

Please outline where this information can be found within the submission (e.g., sections or figure legends), or explain why this information doesn't apply to your submission:

No power analysis was run for this study. Description on how the numerosity of the sample sizes was obtained in the Main Experiment is provided in the "Methods" section:

"Because this was an exploratory experiment, we recruited an overall of 120 participants, organized in four groups of 30 subjects." ("Methods" section; "Experiment 1" subsection; "Participants" sub-subsection).

"For this experiment, 28 experienced medical students (EMS) enrolled to the 5th and 6th year of medicine at the University of Geneva, responded to our recruitment call. Two of them were excluded, due to [...]. Therefore, the final sample of EMS was composed by 26 participants (9 males, age= 24.15 ± 1.40). EMS participants were associated with a matched control group of 26 students not enrolled in medical-related faculties (13 males, age= 23.73 ± 4.11)." ("Methods" section; "Experiment 2" subsection; "Participants" sub-subsection).

Replicates

- You should report how often each experiment was performed
- You should include a definition of biological versus technical replication
- The data obtained should be provided and sufficient information should be provided to indicate the number of independent biological and/or technical replicates
- If you encountered any outliers, you should describe how these were handled
- Criteria for exclusion/inclusion of data should be clearly stated
- High-throughput sequence data should be uploaded before submission, with a private link for reviewers provided (these are available from both GEO and ArrayExpress)

eLife Sciences Publications, Ltd is a limited liability non-profit non-stock corporation incorporated in the State of Delaware, USA, with company number 5030732, and is registered in the UK with company number FC030576 and branch number BR015634 at the address Westbrook Centre, Milton Road Cambridge CB4 1YG, UK | March 2019

Please outline where this information can be found within the submission (e.g., sections or figure legends), or explain why this information doesn't apply to your submission:

In this study the main paradigm was tested twice, once in a population of 120 participants in Experiment 1, and a second time in a population of 52 participants in Experiment 2. This information is available throughout the manuscript. E.g.:

"In Experiment 1 we recruited 120 participants, organized as 30 Controls, 30 YMS, 30 IMS and 30 EMS. [...] In Experiment 2, we repeated the same paradigm by focusing specifically on 52 participants: 26 Controls and 26 EMS." ("Results" section; "Pain Expressions Rating" subsection).

In the Results section, and in the figures, we privilege reporting replications across different experiments together. See Figures 2 & 4. See also a specimen paragraph:

"In both experiments, the analysis of Reappraisal revealed a main effect of Protagonist (Exp 1: b = 0.13, $F_{(1,59.45)}$ = 60.21, p < .001; Exp 2: b = 0.07, $F_{(1,31.62)}$ = 5.87, p = .021) and of MPs (Exp 1: b = 0.12, $F_{(1,88.42)}$ = 21.40, p < .001; Exp 2: b = 0.10, $F_{(1,55.58)}$ = 12.33, p < .001) feedbacks, revealing that any feedback deviating ~ 1 point in the scale led to an adjustment of ~ 0.1 in the same direction, regardless of whether this feedback was from the Protagonist or the MPs." ("Results" section; "Effect of Feedback" subsection).

Those instances where there was no exact replication, were explicitly mentioned in the manuscript, both in the results and in the discussion.

*"we found that, in Experiment 1, the Reappraisal was associated with a significant MPs*Distrust interaction (Exp 1: b = 0.04, $F_{(1,3756.7)}$ = 6.46, p = .011; Exp 2: b = -0.02, $F_{(1,80.56)}$ = 0.48, p = .492). [...] Unfortunately, we did not replicate this behavioral effect in Experiment 2 (in which a smaller sample size, and only two groups, were collected)."* ("Results" section; "Effect of Distrust" subsection).

"In Experiment 1, participants most doubtful about the pain's authenticity adjusted their response more strongly towards MPs. This effect was not replicated in Experiment 2 (in which we recruited a smaller sample size, and did not include young and intermediate medical students). However, in Experiment 2, Distrust influenced the neural responses [...]" ("Disussion" section; "Distrust influences specific Feedback Processing" subsection).

Also the secondary task (Implicit Association Task), was repeated twice across the study (in an population of 155 subjects in Pilot 1, and in the 52 participants from Experiment 2), with replicated effects. This was explicitly displayed in Figure 2, and mentioned in the results section

"Through an implicit association task (IAT), we found that EMS showed stronger positive disposition towards the category of MPs (relative to non-MPs), an effect which was less pronounced in Controls and less experienced students (see Figure 2A; see also Figure 2D for a similar effect in subjects from Experiment 2).

In our study we report explicitly the inclusion criteria for our groups. E.g.,

"The first group comprehended lay individuals (12 males out of 30, mean age= 25.03 years ± 4.75 Standard Deviation [SD]) who were recruited among different faculties and professions, except those related to medicine, infirmary, dentistry and physiotherapy. The remaining 90 participants (26 males, mean age= 22.56 ± 2.86) were recruited among students enrolled from the 1st to the 6th year of medical faculty at the University of Geneva and Lausanne (1st year=14, 2nd year=16, 3rd year, N = 15; 4th year, N = 15; 5th year, N = 17; 6th year, N = 13)." ("Methods" section; "Experiment 1" subsection; "Participants" sub-subsection).

Finally, in Experiment 2 we encountered two participants who did not comply with the task instructions, and as such were excluded. This is explicitly mentioned in the methods section.

"For this experiment, 28 experienced medical students (EMS) enrolled to the 5th and 6th year of medicine at the University of Geneva, responded to our recruitment call. Two of them were excluded due to non-compliance with the task. Therefore, the final sample of EMS was composed by 26 participants (9 males, age= 24.15 ± 1.40)." ("Methods" section; "Experiment 2" subsection; "Participants" sub-subsection).

In our study we have no genetic/sequence data. We do have neural response evoked from Experiment 2 which are uploaded in the Neurovault repository: https://neurovault.org/collections/9006/

Statistical reporting

- Statistical analysis methods should be described and justified
- Raw data should be presented in figures whenever informative to do so (typically when N per group is less than 10)
- For each experiment, you should identify the statistical tests used, exact values of N, definitions of center, methods of multiple test correction, and dispersion and precision measures (e.g., mean, median, SD, SEM, confidence intervals; and, for the major substantive results, a measure of effect size (e.g., Pearson's r, Cohen's d)
- Report exact p-values wherever possible alongside the summary statistics and 95% confidence intervals. These should be reported for all key questions and not only when the p-value is less than 0.05.

Please outline where this information can be found within the submission (e.g., sections or figure legends), or explain why this information doesn't apply to your submission:

Detailed information on the statistical approached used can be found in the paragraph "Data Analysis" from the section "Methods" of each experiment.

Our group sizes are always N ≥ 26. In our figures we always display individual data-points, superimposed to boxplots/regression lines. In such was the reader can have a direct access of the data distribution underlying our effects.

Furthermore, in our figures results are displayed through boxplots, described in terms of median line, interquartile range box, and full range data whiskers. Likewise, linear regressions are always plot with 95% confidence intervals area. This information is always explicitly mentioned in the legends of each figure. Likewise in the text, we always report explicitly the measures of center and dispersion. E.g.,

"The first group comprehended lay individuals (12 males out of 30, mean age= 25.03 years ± 4.75 Standard Deviation [SD])" ("Methods" section; "Experiment 1" subsection; "Participants" sub-subsection).

Effect sizes of linear models of behavioural and neural activity are reported in terms of parameter estimates (*b* for Linear Mixed Models on behavioural responses; *β* for general linear models on brain responses). Effects' sizes on linear regressions are reported in terms of Spearman's *ρ*.

Finally, in the text p-values are always reported in exact form. In Figures/Tables, however, significant tests are displayed according the following legend "***", "**", and "*" referring to $p < 0.001$, $p < 0.01$ and $p < 0.05$.

(For large datasets, or papers with a very large number of statistical tests, you may upload a single table file with tests, Ns, etc., with reference to sections in the manuscript.)

Group allocation

- Indicate how samples were allocated into experimental groups (in the case of clinical studies, please specify allocation to treatment method); if randomization was used, please also state if restricted randomization was applied
- Indicate if masking was used during group allocation, data collection and/or data analysis

Please outline where this information can be found within the submission (e.g., sections or figure legends), or explain why this information doesn't apply to your submission:

Our grouping factor was based on participants' educational choices. Hence, the use of masking allocation strategies was not relevant for this study.

Participants' recruitment as well as inclusion criteria are indicated in the Methods section "Participants" for both experiments. E.g.,

"The first group comprehended lay individuals (12 males out of 30, mean age= 25.03 years ± 4.75 Standard Deviation [SD]) who were recruited among different faculties and professions, except those related to medicine, infirmary, dentistry and physiotherapy. The remaining 90 participants (26 males, mean age= 22.56 ± 2.86) were recruited among students enrolled from the 1st to the 6th year of medical faculty at the University of Geneva and Lausanne (1st year=14, 2nd year=16, 3rd year, N = 15; 4th year, N = 15; 5th year, N = 17; 6th year, N = 13)." ("Methods" section; "Experiment 1" subsection; "Participants" sub-subsection).

Additional data files ("source data")

- We encourage you to upload relevant additional data files, such as numerical data that are represented as a graph in a figure, or as a summary table
- Where provided, these should be in the most useful format, and they can be uploaded as "Source data" files linked to a main figure or table
- Include model definition files including the full list of parameters used
- Include code used for data analysis (e.g., R, MatLab)
- Avoid stating that data files are "available upon request"

Please indicate the figures or tables for which source data files have been provided:

All the results graph depicted in the paper are based on data, which have been made available through certified repositories. Please see the the section of the paper "Data Availability".

"The behavioral data and script are stored and available at the following link: https://osf.io/qnp6m/. The brain imaging data are stored and available at the following link: https://neurovault.org/collections/9006/."
